# Protective Effects of Human iPS-Derived Retinal Pigment Epithelium Cell Transplantation in the Retinal Dystrophic Rat

**DOI:** 10.1371/journal.pone.0008152

**Published:** 2009-12-03

**Authors:** Amanda-Jayne Carr, Anthony A. Vugler, Sherry T. Hikita, Jean M. Lawrence, Carlos Gias, Li Li Chen, David E. Buchholz, Ahmad Ahmado, Ma'ayan Semo, Matthew J. K. Smart, Shazeen Hasan, Lyndon da Cruz, Lincoln V. Johnson, Dennis O. Clegg, Pete J. Coffey

**Affiliations:** 1 Department of Ocular Biology and Therapeutics, Institute of Ophthalmology, University College London, London, United Kingdom; 2 Center for Stem Cell Biology and Engineering, Department of Molecular, Cellular and Developmental Biology, University of California Santa Barbara, Santa Barbara, California, United States of America; 3 Center for the Study of Macular Degeneration, University of California Santa Barbara, Santa Barbara, California, United States of America; 4 Department of Vitreoretinal Surgery, Moorfields Eye Hospital, London, United Kingdom; University of Oldenburg, Germany

## Abstract

Transformation of somatic cells with a set of embryonic transcription factors produces cells with the pluripotent properties of embryonic stem cells (ESCs). These induced pluripotent stem (iPS) cells have the potential to differentiate into any cell type, making them a potential source from which to produce cells as a therapeutic platform for the treatment of a wide range of diseases. In many forms of human retinal disease, including age-related macular degeneration (AMD), the underlying pathogenesis resides within the support cells of the retina, the retinal pigment epithelium (RPE). As a monolayer of cells critical to photoreceptor function and survival, the RPE is an ideally accessible target for cellular therapy. Here we report the differentiation of human iPS cells into RPE. We found that differentiated iPS-RPE cells were morphologically similar to, and expressed numerous markers of developing and mature RPE cells. iPS-RPE are capable of phagocytosing photoreceptor material, *in vitro* and *in vivo* following transplantation into the Royal College of Surgeons (RCS) dystrophic rat. Our results demonstrate that iPS cells can be differentiated into functional iPS-RPE and that transplantation of these cells can facilitate the short-term maintenance of photoreceptors through phagocytosis of photoreceptor outer segments. Long-term visual function is maintained in this model of retinal disease even though the xenografted cells are eventually lost, suggesting a secondary protective host cellular response. These findings have identified an alternative source of replacement tissue for use in human retinal cellular therapies, and provide a new *in vitro* cellular model system in which to study RPE diseases affecting human patients.

## Introduction

The retinal pigment epithelium (RPE) is a monolayer of cells, residing at the back of the eye between Bruch's membrane and the retina, which is essential for photoreceptor function and survival. Dysfunction and death of RPE has been observed in various human degenerative diseases that lead to blindness, including one of the leading causes of blindness in the western world, aged-related macular degeneration (AMD). The limited benefit of existing clinical and surgical interventions for these diseases[1] has led to increased interest in the development of a cell-based transplantation therapy. Various cell types have been examined for use in RPE cell replacement including immortalized cell lines, such as the human RPE cell line, ARPE19[2], sheets of adult RPE[3], foetal RPE[4], RPE derived from human embryonic stem cells (HESC-RPE)[5]–[10] and many non-RPE cells lines[11]–[15].

Current methods, producing stem cells from adult somatic cells, offer an alternative cell source for transplantation. Induced pluripotent stem (iPS) cells are morphologically identical to embryonic stem cells, display similar gene expression profiles and epigenetic status, and have the potential to form any cell in the body [16]–[18]. iPS cells have been employed to generate cells for the treatment of various diseases including diabetes, cardiovascular disease, sickle cell anaemia, Parkinson's disease and haemophilia[19]–[23]. Meyer et al 2009[24] have recently shown that iPS cells can be differentiated towards retinal cell types whilst a paper by Buchholz et al 2009[25] has shown that human iPS cells can be differentiated into retinal pigment epithelial cells which display functionality *in vitro*. As yet it is unknown whether iPS cells can differentiate into functional replacement cells for use in the treatment of progressive diseases specific to the visual system. Here we examine the potential of human iPS cells to differentiate into fully characterized RPE cells (iPS-RPE). Furthermore we analyse their functionality *in vitro*, and *in vivo* after transplantation of iPS-RPE into the dystrophic RCS rat: a model of retinal dystrophy where the primary defect, originating in RPE cells [26], leads to blindness as a consequence of rod and cone photoreceptor degeneration[27]–[29].

## Materials and Methods

### Derivation of iPS-RPE Cells and Cell Culture

The human induced pluripotent stem cell clone, iPS(IMR90)-3[18], was passaged onto Mitomycin-C inactivated mouse embryonic feeder cells with DMEM/F12 culture medium containing 20% Knock-Out Serum Replacement, 0.1 mM non-essential amino acids, 0.1 mM β-mercaptoethanol and 100 ng/ml zebrafish basic fibroblast growth factor (zfbFGF) on a 6-well plate. Cells were cultured at 37°C in 5% CO_2_ for 6 days after which zfbFGF was omitted to facilitate spontaneous iPS cell differentiation. Pigmented colonies were observed within 4 weeks and allowed to expand for a further 14 weeks, with media changes every 2–3 days. Pigmented cells were enriched by manual dissection of expanded colonies followed by dissociation in 0.05% Trypsin-EDTA. Cells were seeded at a density of 1.2×10^4^ cells/cm^2^ onto gelatin-coated plates with Human foetal RPE medium[30] containing α-MEM, 1 x N1 supplement, 1 x Non-essential amino acid solution, 250 mg/ml taurine, 13 ng/ml Triiodo thyronin (Sigma-Aldrich, Gillingham, UK), 20 ng/ml Hydrocortisone (Sigma), 2mM L-glutamine (Invitrogen, Paisley, UK), and 15% Hyclone heat-inactivated foetal bovine serum (Thermo Scientific, Northumberland, UK), which was replaced daily. Upon cells reaching confluency the serum concentration of the medium was reduced to 5% and the media replenished twice weekly. Subsequent passages were performed using Trypsin-EDTA dissociation. Cells were then plated onto gelatin-coated flasks, dishes and transwell membranes.

### Characterization of Cells

#### Immunocytochemistry

iPS-RPE cells or sheets of cells were fixed for 30 min with 4% paraformaldehyde in 0.1 M phosphate buffer. Sheets of cells were washed and scraped off the dish using a cell scraper, cryoprotected in 30% sucrose in PBS and rapidly frozen in OCT (Tissue Tec ®). Sections (14 µm) were cut onto charged glass slides (VWR). Cells on dishes, or sections were blocked and incubated with appropriate combinations of primary antibodies as described previously[9]. The following primary mouse monoclonal antibodies were used: MITF (1∶30, Abeam), RLBP1 (CRALBP, 1∶1000, Affinity Bio reagents); BEST1 (1∶1000, Millipore); RPE65 (1∶500, Millipore); PMEL17 (1∶100 Dako); KRT8 (1∶2000, Millipore); Na^+^/K^+^ATPase (ATP1B1 - 1∶100, Abcam). Primary rabbit polyclonals used were OTX1/2 (1∶500, Millipore); PAX6 (1∶300, Covance); Ki67 (1∶2000, Vector labs); ZO1 (1∶50 Zymed) and Type IV Collagen (1∶100, Morwell Diagnostic Biosciences). Secondary antibodies were donkey anti-mouse or anti-rabbit FITC or TRITC (1∶200, Jackson ImmunoResearch Labs Inc.). Nuclei were visualised with DAPI (1∶5000, 4′6-diamindino-2-phenylindole dihydrochloride, Sigma). Specificity of staining was tested by omission of the primary antibodies. Images were obtained using a Zeiss confocal microscope with Nomarski optics and analysed with LSM Image Browser software. TIFF images were imported into the ImageJ[31] processing program to produce movies.

#### Electron microscopy

iPS-RPE cells were grown in gelatin-coated transwells and then fixed in Karnovsky's fixative (1% paraformaldehyde and 3% glutaraldehyde in 0.1 M cacodylate buffer). Cells plus membrane were excised from the transwell and post-fixed in 1% osmium tetroxide. After dehydration through a graded series of alcohols and epoxypropane, cells were transferred to resin (Araldite Cy212, Agar Scientific) and polymerized at 60°C. Ultrathin sections were stained with uranyl acetate and lead citrate prior to examination in a Jeol 1010 electron microscope.

#### Reverse transcription-Polymerase Chain Reaction (RT-PCR)

RNA was extracted from iPS-RPE cells using TriZol reagent (Invitrogen) and DNA removed using RQ1-RNase Free DNase. cDNA was then synthesized from 3 µg of RNA using Superscript III Reverse transcriptase (Invitrogen). Gene expression was analysed by amplifying 1 µl of cDNA synthesis product in a PCR Mastercycler® (Eppendorf, Cambridge, UK) using Go Taq Polymerase (Promega) in a reaction containing 0.2 µM of gene-specific primers (Eurofins MWG Operon, Ersberg, Germany). For primer sequence and annealing temperature (Ta) see Table S1. The PCR cycle parameters consisted of an initial denaturation at 95°C for 2 min followed by 35 cycles of denaturation at 95°C for 30 s, annealing at Ta °C for 30 s, and elongation at 72°C for 30 s. PCR was completed with a final elongation step at 72°C for 5 min. PCR products were resolved on a 2% agarose gel alongside a 100 bp DNA ladder (Promega).

#### Quantitative PCR

Total RNA was isolated from iPS, iPS-RPE and foetal RPE cell cultures using the Qiagen RNeasy Mini Kit (Qiagen, Valencia, CA). Contaminating genomic DNA was digested using RQ1 RNase-free DNase (Promega, Madison, WI) and the RNA purified again with the RNeasy Kit. cDNA was synthesized from 1 µg of RNA using the iScript cDNA Synthesis Kit (Bio-Rad, Hercules, CA). Quantitative real-time PCR was carried out on a Bio-Rad MyIQ Single Color Real-Time PCR Detection System using the SYBR Green method. Triplicate 20 µl reactions were run in a 96 well plate with half of the cDNA synthesis reaction used per plate. Primer specificity was assessed by melting temperature analysis, gel electrophoresis and direct sequencing (Iowa State DNA Facility, Ames, IA). Data was normalized to the geometric mean of glyceraldehyde phosphate dehydrogenase (*GAPDH*), peptidylprolyl isomerase A (*PPIA*), hydroxymethylbilane synthase (*HMBS*) and glucose phosphate isomerase (*GPI*). For primer sequence and annealing temperature (Ta) see Table S1.

#### Western blotting

iPS-RPE cells were collected on ice in lysis buffer (10 mM HEPES, 1% Triton, 150 mM KCl, 1 mM PMSF, 10 ng/ml leupeptin, 1 mM DTT, 50 ng/ml aprotinin, 10 mM NaF, and 100 µM sodium vanadate) and incubated for 30 min on a tube rotator at 4°C. Cell debris was removed from the sample by centrifugation at 13,000 rpm for 30 min at 4°C and the supernatant diluted in sample buffer. Proteins were denatured at 95°C for 5 min, separated on a SDS-PAGE gel alongside a Dual Color Precision Plus Protein Standard (Biorad) and transferred to Hybond PVDF membrane (GE Healthcare Life Sciences, Buckinghamshire UK). Membranes were blocked for 2 hours in 10% milk in PBS-0.05% Tween-20 and incubated overnight in 10% milk containing primary antibodies raised in Mouse: RPE65 (1∶2000, Chemicon); RLBP1 (CRALBP, 1∶2000, Affinity BioReagents, CO, USA); KRT8 (1∶2000, Chemicon); PEDF (1∶500, Chemicon); PMEL17 (1∶100, Dako); Rabbit: MERTK (1∶500, Abcam); FAK (0.1 µg/ml, Stratech) and MITF (1∶500, Chemicon). Membranes were washed in PBS-0.05% Tween-20, incubated for an hour with secondary HRP-conjugated antibodies, then washed and incubated in LumiLight western blotting solution (Roche Products Ltd., Welwyn Garden City, UK). Proteins were detected by exposure to autoradiographic film.

#### In vitro phagocytosis assay

Photoreceptor outer segments (POS) were isolated from freshly slaughtered porcine eyes using a continuous sucrose gradient as previously described[8], [32]. POS were then labelled with AlexaFluor ® 488 Dye (Invitrogen) in 0.1 M sodium bicarbonate/5% sucrose in a light-tight microcentrifuge tube for 1 h at room temperature. Labelled outer segments were washed, resuspended in human foetal RPE medium and seeded onto iPS-RPE cells cultured on gelatin-coated 35 mm dishes. Cells were incubated at 37°C in 5% CO_2_ for 20 h. External fluorescence was removed by treatment with trypan blue for 10 min. Cells were then washed in PBS and processed for immunocytochemistry. iPS-RPE were also incubated with porcine retina explants in an *in vitro* preparation previously described[8]. Briefly, iPS-RPE were cultured on gelatin-coated culture plate inserts. Fresh porcine retinal tissue was dissected, orientated on a 0.45 mm filter with the photoreceptor cell surface uppermost and placed on top of the iPS-RPE monolayer so that outer segments were adjacent to the RPE. The explant model was incubated in Human foetal RPE medium at 37°C in 5% CO_2_ for 3 or 12 hours. The iPS-RPE plus retina was fixed and processed for electron microscopy as above.

#### Transplantation

Host animals were 22–23 day old dystrophic (rdy^−^/p^+^) and non-dystrophic control, pigmented (rdy^+^/p^+^) Royal College of Surgeons (RCS) rats, which were maintained in a 12 h light/dark cycle. Food and water were given *ad libitum* and all procedures were carried out in accordance with the UK Home Office regulations under the Animals (Scientific procedures) Act 1986. The water contained 210 mg/l ciclosporin (Sandoz, Camberley, UK) and was given to all animals (experimental and controls) 48 h prior to surgery and throughout the duration of the experiment. Rats were anaesthetized with a mixture of medetomidine hydrochloride and ketamine. iPS-RPE cells were suspended in α-MEM (Sigma) at a density of 5×10^4^ cells per microlitre and 2 µl were injected into the subretinal space of the left eye (N = 10) between the host RPE and photoreceptor cells, using a 30-gauge needle attached to a Hamilton syringe. Control sham injections containing an equivalent volume of α-MEM medium only were injected into the dorsal subretinal space in an identical manner (N = 6). Four further dystrophic and 4 non-dystrophic (normal control) rats remained as unoperated controls. Animals were tested for visual function 13 weeks post-transplantation (16 weeks of age), a time point where sham surgery no longer exerts an effect on photoreceptor rescue[11]. Termination was then achieved by anaesthetic overdose and transcardiac perfusion of PBS followed by 4% paraformaldehyde. Two of the iPS-RPE grafted animals were sacrificed 8 days post-surgery for intermediate anatomical assessment. Following perfusions, eyes were either excised, cryoprotected, frozen and cut as described for immunocytochemistry above or the retina was removed intact as a whole-mount preparation.

#### Functional assessment and anatomy

Transplant efficacy was assessed in two ways: firstly by optokinetic testing whereby an animal that can see will involuntarily move its head in response to a moving stimulus, and secondly, by examining light-induced c-Fos activation in the host retina. The induction of c-Fos expression in the inner nuclear layer (INL) and ganglion cell layer (GCL) of the retina in response to light is an indication of functioning retinal circuitry. The location of transplanted human cells, their expression profile and ability to phagocytose rod photoreceptor material was examined *in vivo* using immunohistochemistry.

#### Optokinetic testing

We used the head-tracking response to moving vertical lines to examine visual acuity in the RCS rat 13 weeks following surgery. The method for assessing head-tracking (optokinetic response) has been reported previously[2]. Briefly, animals were placed into a circular container and exposed to a rotating stimulus consisting of vertical black and white lines of varying widths, subtending the spatial frequencies of 0.312, 0.25, and 0.5 cycles/degree which rotate clockwise (to test acuity in the left eye) or anticlockwise (to test acuity in the right eye). Each eye was tested twice over a 60 second period. Animals that could see made a well-defined “head-tracking” movement, following the moving vertical lines. Testing was performed over three consecutive days and the results were videotaped. The time spent head-tracking over each 60 second period was measured blind off-line by a single observer.

#### Statistical analysis

Head-tracking data was analysed using SPSS, version 12.0.1. Two-way repeated-measures ANOVA was performed to compare the optokinetic response between the clockwise and counterclockwise directions as a function of spatial frequency. *Post hoc* t-tests using a Bonferroni correction were used to establish the spatial frequencies at which the difference between both directions was significantly different. The effect of cell transplantation treatment on the optokinetic response was compared as a function of spatial frequency by using two-way ANOVA with treatment as an independent-measures factor and spatial frequency as a repeated-measures factor. Data are shown as mean ± SEM and P<0.05 was considered to be statistically significant.

#### Functional anatomy

The expression of the immediate early gene c-Fos was used to assess integrity of retinal function following iPS-RPE transplantation. Following optokinetic assessment, animals were dark-adapted overnight and then either sacrificed in darkness (using a dim red light) or following 90 minutes of constant illumination (white light at 250 µW/cm^2^). Retinal tissue (whole-mounts or sections) were stained with rabbit anti-c-Fos (1∶5000, Calbiochem).

#### Immunohistochemistry to identify transplanted human cells

The antibodies listed above in the immunocytochemistry section were also used on eye sections together with a cocktail of antibodies generated against human-specific markers (HSM) to identify human cells (the Oka blood group antigen, mouse TRA-1-85, 1∶10 (a kind gift from Peter Andrews, University of Sheffield, Sheffield, UK) together with mouse human nuclear antigen, 1∶1000, Millipore). Additionally, human-specific mouse anti-Ki67 (1∶50, Dako) revealed any human cells in active phases of the cell cycle. Where mouse monoclonal antibodies were used to probe for RPE specific markers in iPS-RPE cells *in vivo* (e.g. anti-RLBP1 and anti-PMEL17), the human identity of target cells was confirmed by subsequent re-probing of sections with human specific markers (HSM) as described previously[9]. Immunocyctochemistry was also used to assess phagocytosis of host outer segments in vivo. Phagocytosis of photoreceptor outer segments was defined by the inclusion of rhodopsin-labelled material within the TRA-1-85-bordered intracellular compartment of grafted human iPS-RPE cells.

## Results

### Differentiation of iPS into RPE Cells

**Figure 1:**
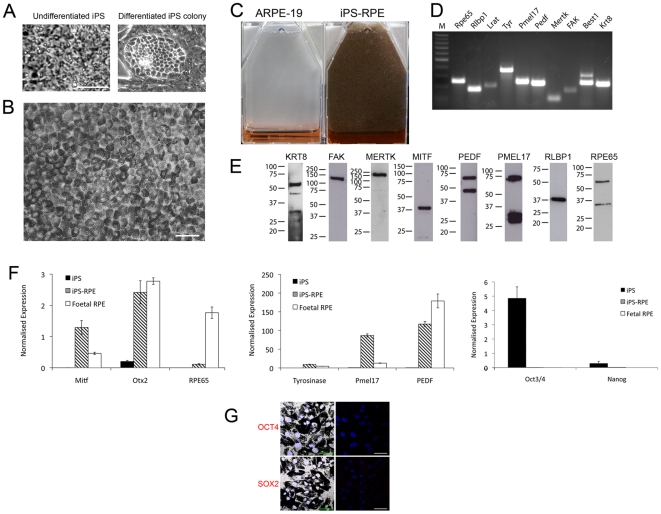
Human induced pluripotent stem cells differentiate into retinal pigment epithelial cells. (A) Photomicrographs showing undifferentiated (left) and differentiating iPS cells (right). (B) iPS-RPE cells form a pigmented monolayer in culture with typical RPE cell cobblestone appearance. (C) Comparison of pigmentation observed in T25 flasks of confluent ARPE-19 cells and iPS-RPE cells. (D) PCR amplification of classic RPE cell markers in iPS-RPE cells. A 100 bp DNA ladder was applied to the gel as an amplicon size reference. (E) Western blot analysis of iPS-RPE protein expression using antibodies against a panel of RPE cell markers. A protein standard was used on each Western blot to determine the correct molecular weight of proteins. (F) Quantitative PCR analysis of RPE and iPS gene expression in iPS, iPS-RPE and foetal RPE cDNAs. (G) iPS reprogramming proteins are reduced after differentiation to iPS-RPE. OCT4 and SOX2 expression is undetectable in iPS-RPE cells. Combined confocal and Nomarski image of iPS-RPE cells are shown on the left with confocal channels to the right. OCT4 and SOX2 (red channel) and DAPI stained nuclei are blue. Scale bars: A, 20 µm; B and G, 50 µm.

iPS cells, derived from the IMR-90 human foetal lung fibroblast cell line[18], were cultured in stem cell medium lacking bFGF to encourage the spontaneous differentiation of cells (Fig. 1A). This led to the appearance of pigmented colonies within 4 weeks. Colonies were allowed to expand for a further 14 weeks before we enriched for pigmented cells by manual dissection of expanded pigmented colonies followed by cell dissociation. Cells were cultured in human foetal RPE medium[30] where, within 13 days, they formed a pigmented monolayer with characteristic RPE cobblestone appearance similar to that observed in human post-mortem RPE sheets and HESC-derived RPE cells[9] (Fig. 1B). The enrichment method employed produced an almost homogeneous population of iPS-RPE cells at passage 2 in a 25 cm^2^ tissue culture flask (Fig. 1C) with no evidence of cell multi-layering. The appearance of dome-shaped blisters suggested that fluid transport from the apical to basal surface of cells might be occurring.

### Characterisation of iPS-RPE Cells

The appearance of RPE cell morphology was associated with the expression of a panel of classic RPE genes and proteins required for retinoid recycling (RPE65, LRAT, RLBP1), phagocytosis (FAK and MERTK) and melanogenesis (Tyrosinase, PMEL17 and MITF). Cells also expressed the anti-neovascular agent/neurotrophic factor PEDF and KRT8, an epithelial keratin associated with RPE cell proliferation[9] (Fig. 1D and 1E). The increase of RPE cell markers in iPS-RPE was accompanied by the down-regulation of the iPS reprogramming molecules OCT4, SOX2 and NANOG expression (Fig. 1F and 1G), indicative of differentiation away from the iPS cell phenotype.

**Figure 2:**
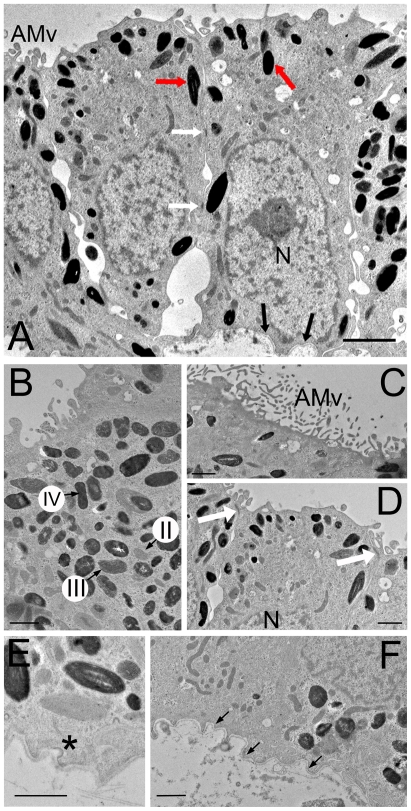
Human iPS-RPE cells are polarized and display classic RPE cell morphology. (A) Electron micrograph of an iPS-RPE cell monolayer. Human iPS-RPE are pigmented cuboidal epithelial cells with cytoplasmic polarization. Indicated are apical microvilli (AMv), melanin-containing melanosomes (red arrows), the basal nucleus (N), desmosomes (white arrows) and basal lamina (black arrows). (B) Densely packed melanosomes: stages II, III and IV of melanosome maturation are labelled. (C) Microvilli (AMv) extend out from the apical surface of iPS-RPE. (D) Adherens junctions (white arrows) between cells are in the apical portion of the cell, whilst the nucleus (N) is basal. (E) Coated pits (asterisk) are found throughout the plasma membrane. (F) iPS-RPE produce their own basal lamina (indicated by black arrows). Scale bars: A, 2 µm; B-F, 1 µm.

**Figure 3:**
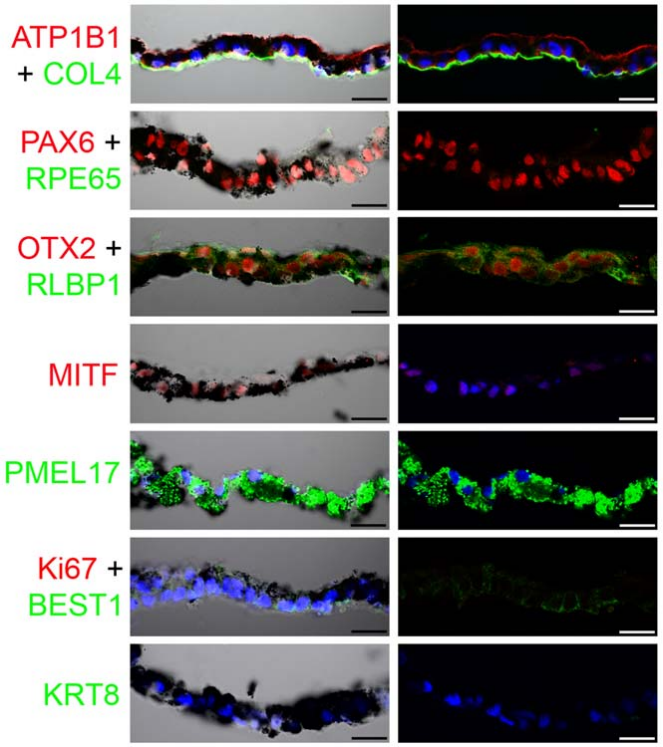
Immunocytochemical localization of RPE cell specific proteins in sectioned sheets of iPS-RPE cells. The left column shows combined Nomarski and confocal images of the pigmented iPS-RPE cell sheet sections; adjacent are images of immunolabelling only. Protein staining is indicated by the colour of the text (red or green) and DAPI stained nuclei are blue. Scale bars: All 50 µm.

Ultrastructurally, melanosomes, responsible for the pigmentation normally observed in RPE cells, were clearly observed within iPS-RPE (Fig. 2A and 2B). Akin to human RPE, the cells were highly polarized with basal nuclei, apical microvilli and adherens junctions (Fig. 2C and 2D). Coated pits, associated with endocytosis, were also present on iPS-RPE cells (Fig. 2E). Cells had secreted their own basal lamina (Fig. 2F), which was positive for the extracellular matrix protein, Collagen IV (COL4) (Fig. 3). In sections through the iPS-RPE monolayer we observed apical expression of Na^+^/K^+^ ATPase (ATP1B1). PAX6, OTX2 and MITF, transcription factors involved in RPE cell development, were localised in the nucleus, whilst RLBP1, PMEL17, and BEST1 were expressed cytoplasmically. Importantly, differentiated cells were negative for RPE cell de-differentiation marker, KRT8[9] and a marker of the active phase of cell cycle, Ki67, indicating cells were no longer proliferative (Fig. 3).

### Functional Assessment of iPS-RPE *In Vitro*

**Figure 4:**
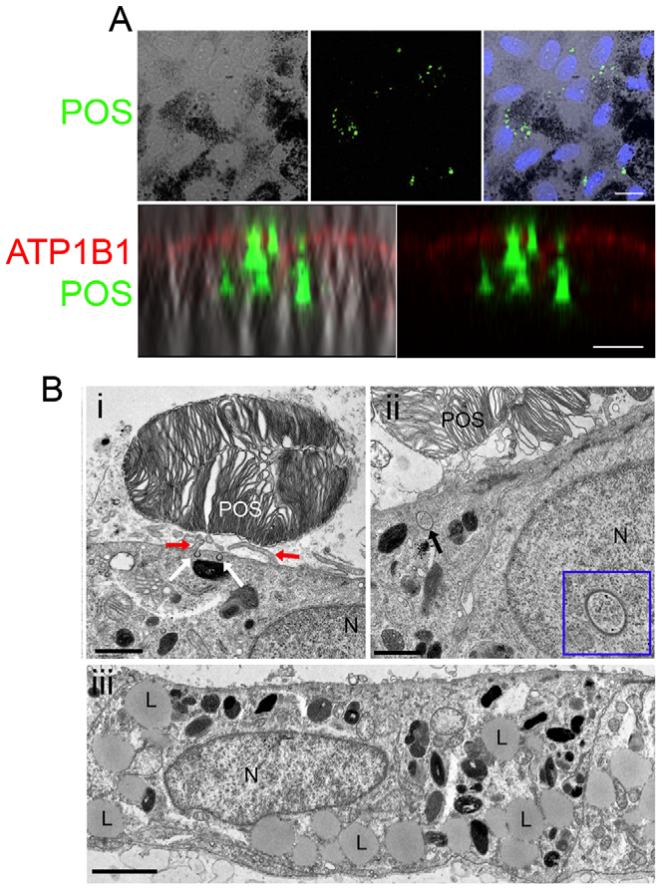
iPS-RPE cells phagocytose photoreceptor outer segment material *in vitro*. (A) Confocal images showing phagocytosis of isolated FITC-labelled porcine photoreceptor outer segments (POS – green) by iPS-RPE in culture. The nuclei are stained with DAPI (blue). Internalization of POS is observed in a single optical y-axis projection (<1 µm) of pigmented iPS-RPE cells labelled with the apical cell surface marker ATP1B1 (red). (B- i) Electron microscopy image of a porcine photoreceptor outer segment (POS) adjacent to an iPS-RPE cell following 3 hours co-culture with a porcine retina explant. iPS-RPE apical microvilli (red arrows) and coated vesicles (white arrows) are observed proximal to the porcine POS. (ii) Internalized coated pits (black arrow and enlarged inset in blue box) are seen within the cytoplasm of iPS-RPE cells co-cultured with POS. (iii) Lipid deposits (L), a sign of late stage POS phagocytosis, are observed within the iPS-RPE cytoplasm after 12 h co-culture. Scale bars: A, Upper panel 20 µm and lower panel 10 µm; i, iii, 2 µm and ii, 1 µm.

We used phagocytosis assays to assess the functional potential of cells. iPS-RPE were able to phagocytose fluorescently labelled porcine photoreceptor outer segments (POS) in co-culture. Confocal microscopy through cells labelled with the apical marker ATP1B1 demonstrated that POS are internalized by iPS-RPE (Fig. 4A and Movie S1). iPS-RPE cells were also able to ingest and digest photoreceptor material from porcine retinal explants. The apical surface of cells envelop the POS (Fig. 4Bi) and internalized coated pits are seen after 3 hours co-culture (Fig. 4Bii), with end-stage lipid deposits observed after 12 hours (Fig. 4Biii).

### *In Vivo* Assessment of iPS-RPE Cell Morphology and Phagocytosis After Transplantation into the RCS Rat

**Figure 5:**
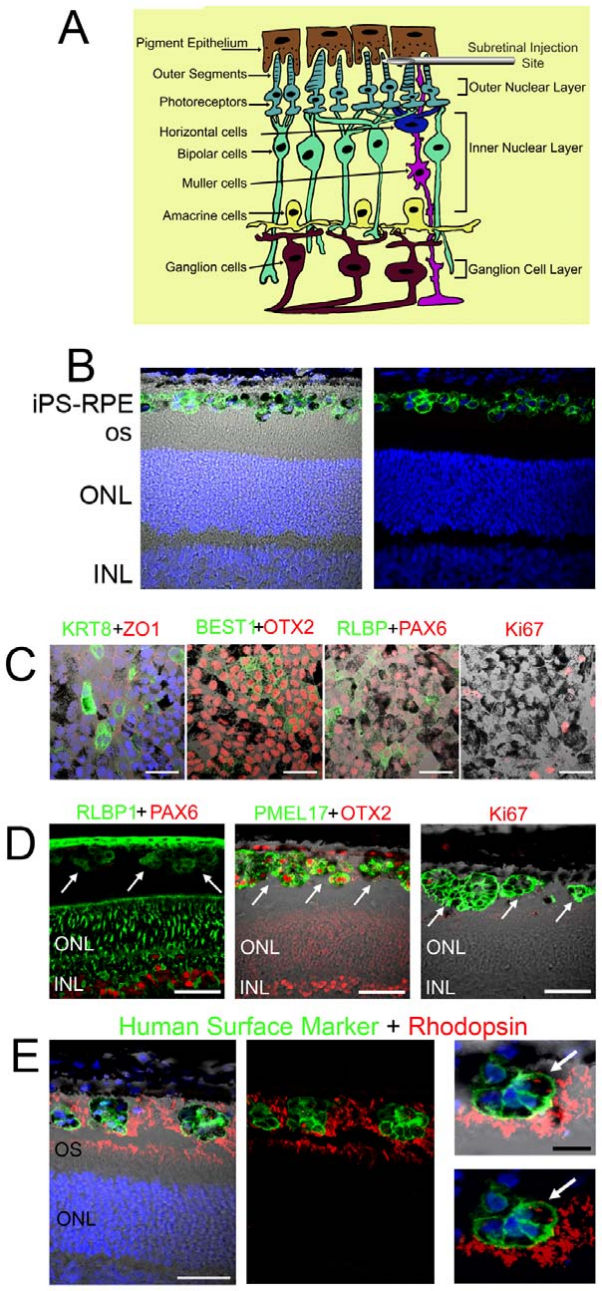
iPS-RPE maintain RPE cell markers and phagocytose host photoreceptor outer segment material following transplantation into the subretinal space of dystrophic RCS rats. (A) A schematic of retinal cell organisation. The nuclear layers are indicated. iPS-RPE cells were injected into the subretinal space between the host RPE and photoreceptor cells (B) A layer of iPS-RPE cells in the subretinal space of the dystrophic RCS rat 20 hours following transplantation. (C) Retention of RPE markers by iPS-RPE cells *in vitro* after dissociation, re-plating and culturing for 8 days. Protein staining is indicated by the text colour. (D) Expression of the same RPE cell markers is maintained *in vivo* by iPS-RPE (white arrows) 8 days following transplantation into the subretinal space of the RCS rat. (E) Rhodopsin-positive material (red) is present within the cell membrane of human specific marker (HSM)-labelled iPS-RPE (green) 8 days post-transplantation and in the tips of the host outer segment (OS) layer. DAPI (blue) stains nuclei. Indicated are the outer and inner nuclear layer (ONL and INL respectively) of the retina. Scale bars: B-D, 50 µm; E, 50 µm and 20 µm in magnified view.

In order to test the therapeutic efficacy of iPS-RPE, we transplanted cells into the subretinal space of RCS dystrophic rats (Fig. 5A). After 20 h we observed a layer of pigmented cells within the subretinal space of the RCS rat, which were Ki67 negative. The origin of the cells was confirmed by staining with HSM (Fig. 5B). We compared the expression of RPE cell markers in transplanted cells *in vivo* against cells re-plated and cultured in parallel. After 8 days in culture, the re-plated iPS-RPE cells were fixed and stained for RPE-associated markers (Fig. 5C). Cells maintained the expression profile they had displayed prior to grafting and had largely exited active phases of the cell cycle (Ki67 negative). At the comparable time point *in vivo*, iPS-RPE cells were observed in the subretinal space and could be clearly identified with the aid of HSM staining (Fig. 5D). At 8 days iPS-RPE were no longer present as a layer, and instead formed cell boluses. However, the transplanted cells maintained expression of RPE markers such as RLBP1 and OTX2, with only occasional cells positive for Ki67. The *in vivo* expression of RPE65 was not detectable by immunocytochemistry. Grafted human cells expressed PMEL17 *in vivo* and could down-regulate Pax6 in the subretinal space, a hallmark of terminally differentiated RPE (Fig. 5D). The pattern of staining displayed by transplanted iPS-RPE cells *in vivo* was similar to that reported recently for HESC-RPE grafted into the dystrophic RCS rat retina[9]. Importantly, akin to HESC-RPE *in vivo*, cells at the outside edge of the iPS-RPE cell bolus could phagocytose photoreceptor outer segments from the RCS rat, as indicated by the presence of rhodopsin-positive photoreceptor material within the cellular membrane of iPS-RPE labelled with HSM (Fig. 5E).

### Assessment of Visual Function in the Dystrophic RCS Rat Following iPS-RPE Transplant

**Figure 6:**
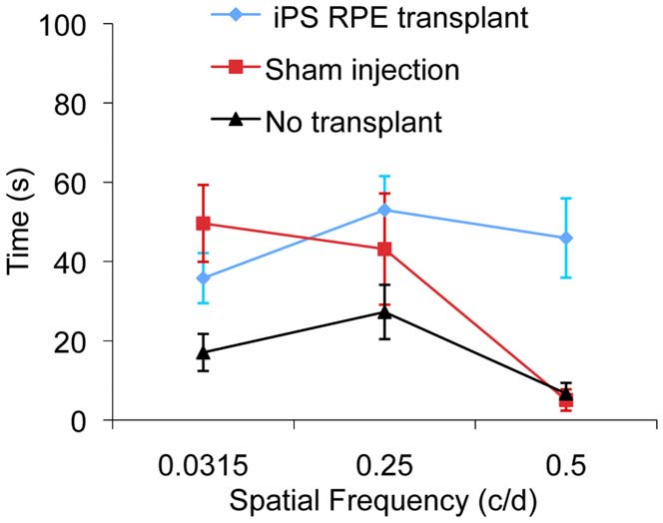
Preservation of visual function following iPS-RPE transplantation into the RCS rat. (A) Preservation of optokinetic head-tracking response to a rotating vertical stimulus in 16-week-old RCS dystrophic rats following transplantation of iPS-RPE. Mean visual acuity (±S.E.M.) of the transplanted eye versus control sham-injected eye and non-transplanted dystrophic eye. Spatial frequency is indicated in cycles per degree (c/d).

**Figure 7:**
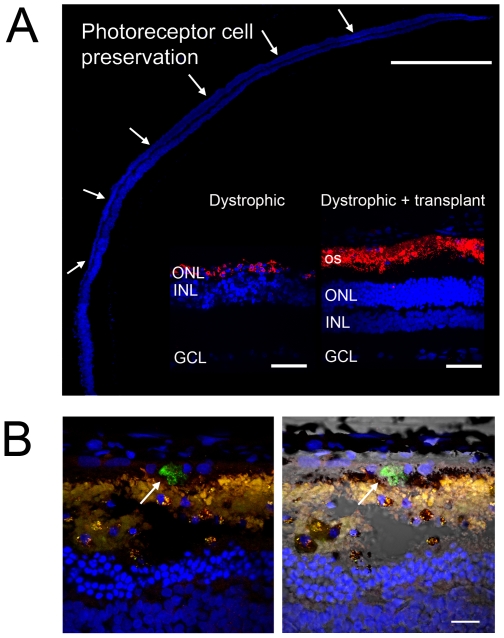
Preservation of the photoreceptor cell layers after transplantation of iPS-RPE. (A) Extensive preservation of the nuclear photoreceptor layers in the dorsal retina of the dystrophic RCS rat 13 weeks following transplantation of iPS-RPE cells (DAPI stained nuclei). Inset shows higher resolution confocal images of photoreceptor cell nuclear layers (DAPI blue) and rhodopsin expression (red) in the dystrophic control (left inset) and dystrophic with iPS-RPE transplant (right inset) RCS rat. (B) Confocal images showing HSM-positive staining (green, indicated with white arrow) within the subretinal space 13 weeks post-transplantation. Scale bars: A 500 µm; inset, 50 µm; B 50 µm.

We used the head-tracking response to assess the visual function of RCS dystrophic rats 13 weeks after receiving a subretinal iPS-RPE transplantation in one eye only (Fig. 6 and Movies S2 and S3). Preservation of the higher spatial frequency (0.5 c/d) monocular optokinetic head-tracking response was associated with the iPS-RPE transplanted eye when compared with the sham-injected eye (ANOVA, F (1,14) = 1.753, P<0.05; Post hoc test at 0.5 c/d p = 0.01) and non-transplanted (ANOVA, F(1,9) = 48.8, P<0.001. Post hoc t-test; t(9) = 1.61, p<0.05 for all spatial frequencies). Conservation of visual acuity in the iPS-RPE transplanted eyes was associated with the preservation of photoreceptors in the host outer nuclear layer (ONL – Fig. 7A), identified by the expression of rhodopsin in the outer segments of photoreceptors (Fig. 7A inset, Dystrophic+transplant). At this age, in the RCS dystrophic rat, the ONL is normally reduced to a single layer of cells with an autofluorescent debris zone (Fig. 7A inset, Dystrophic) [9], [33].

### Survival of iPS-RPE *In Vivo* After Transplantation into the Dystrophic RCS Rat

**Figure 8:**
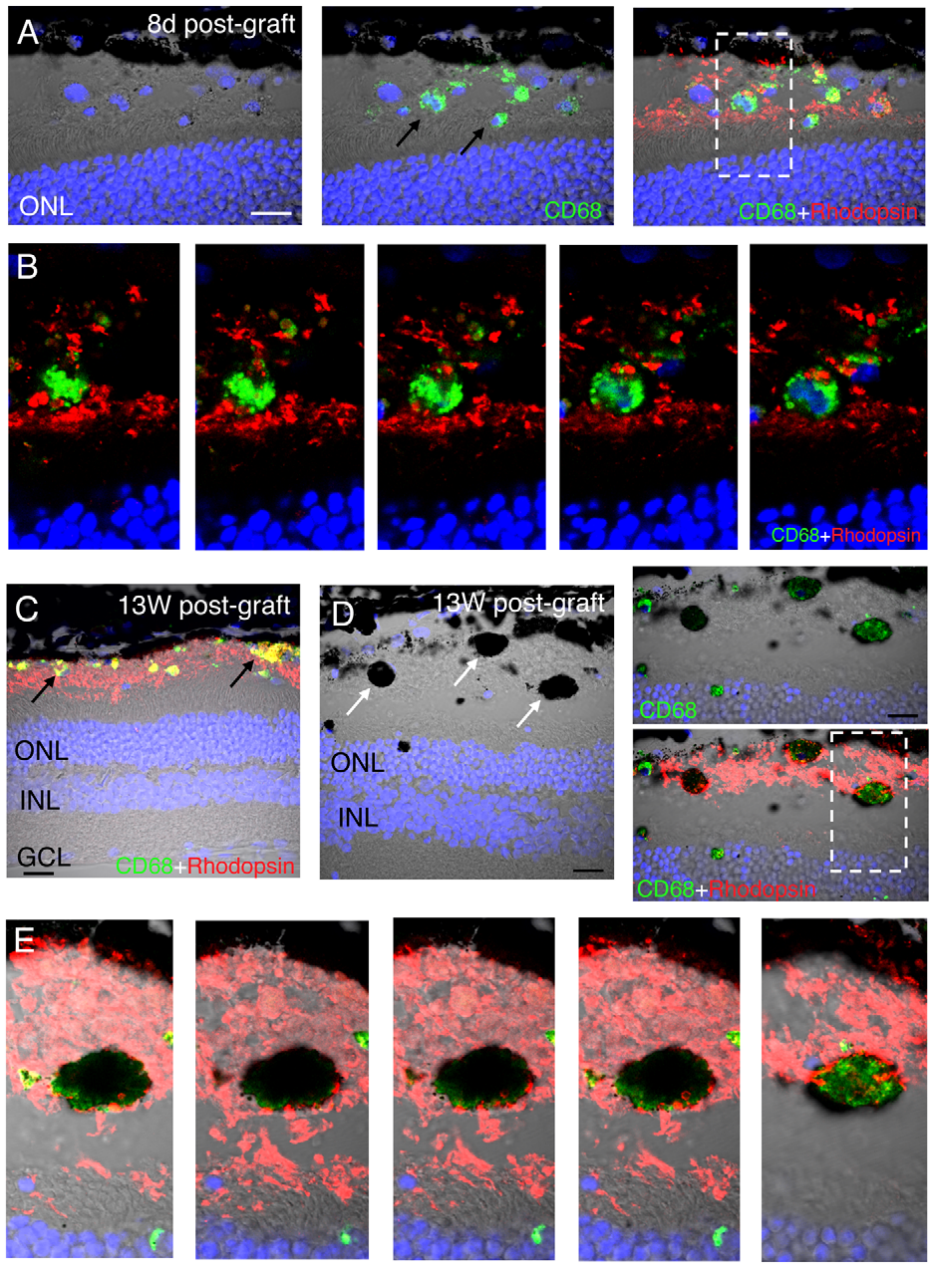
Macrophages are present in the subretinal space of the dystrophic RCS following transplantation of iPS-RPE cells. (A) Coronal confocal projection showing confocal channels and Nomarski images of iPS-RPE transplanted eye containing non-pigmented CD68-positive cells (green) 8 days following transplantation. The white box contains a CD68-positive cell with rhodopsin-positive cytoplasmic inclusions (red) which is magnified and shown in serial confocal slices in B. (C) Co-labelling of non-pigmented CD68 and rhodopsin-positive cells at 13 weeks post-graft. Note the presence of an intact outer segment and outer nuclear layer at this stage. (D) Large pigmented cells are observed in the subretinal space at 13 week post-graft. The white box indicates a CD68-positve cell containing rhodopsin-positive inclusions, shown magnified and in serial confocal slices in (E). Scale bars: All 20 µm.

Even though the ONL was preserved 13 week post-transplantation, there was little evidence of surviving iPS-RPE cells within the subretinal space by this time. Occasional HSM-positive material could be detected in the subretinal space (Fig. 7B), but the lack of a defined membrane and the absence of DAPI staining suggest that these cells were not viable. However, we did find host cells positive for the monocyte/macrophage marker CD68 within the sub-retinal graft site, which did not stain for HSM. At 8 days these cells had a clear appearance (Fig. 8A), and rhodopsin-positive material was found within the CD68-expressing cells (Fig. 8B). At 13 weeks, two distinct CD68-positive cell populations were observed, one type was small and non-pigmented (Fig. 8C) and the other consisted of large highly pigmented cells (Fig. 8 D). Both these cell types contained rhodopsin-positive material (Fig. 8C–E).

**Figure 9:**
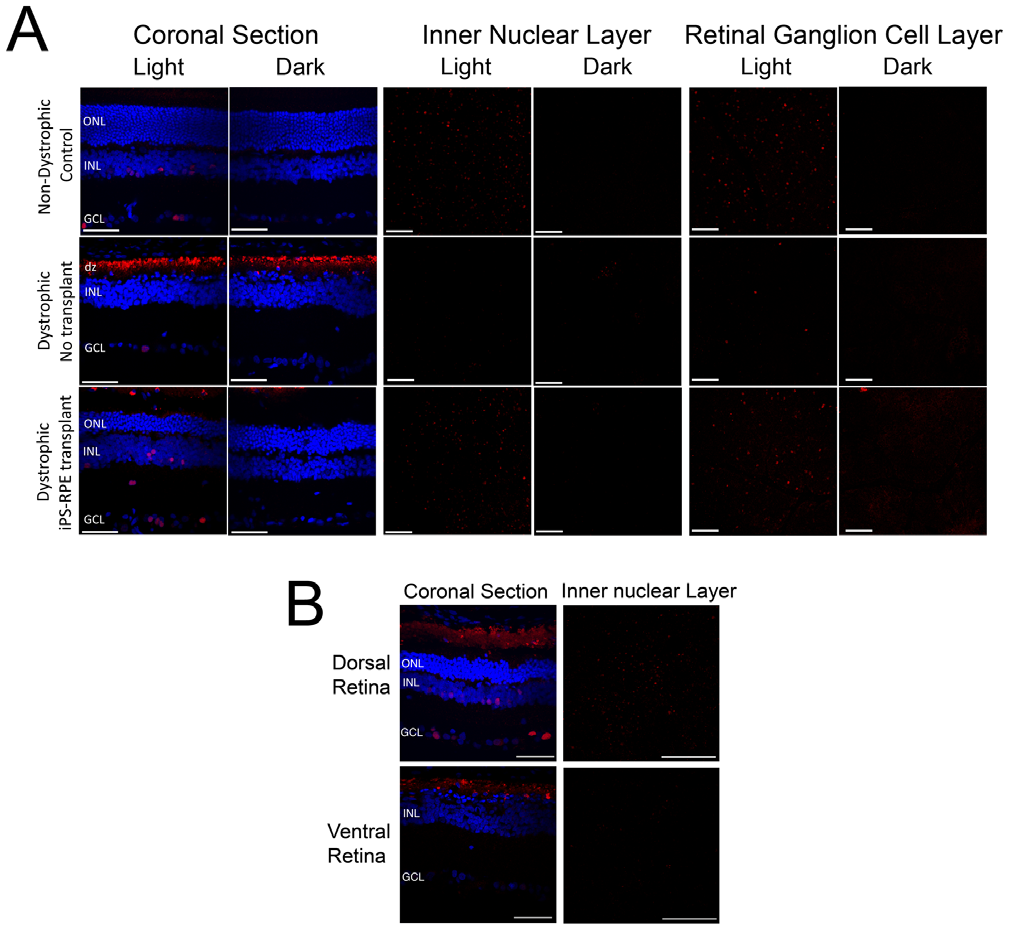
iPS-RPE transplantation preserves the light induced c-Fos response in the RCS dystrophic rat retina. (A) Age-matched non-dystrophic control, dystrophic no-transplant control and dystrophic rats with iPS-RPE grafts were dark-adapted overnight and sacrificed in the dark or after 90 min exposure to white light (250 µW/cm^2^). Coronal sections through the retina of dystrophic transplanted, dystrophic non-transplanted and non-dystrophic control RCS rats showing the inner and outer nuclear layers (ONL and INL respectively) and the ganglion cell layer (GCL). Preservation of ONL 13 weeks after iPS-RPE transplant correlates with preservation of the light-induced c-Fos expression (red) in coronal sections of the retina and in representative dorsal whole-mount preparations of the inner nuclear and ganglion cell layers. Note absence of activity in darkness and responsivity to light in the normal and transplanted eyes. c-Fos positive cells in the ganglion cell layer of unoperated RCS eyes match the distribution expected for intrinsically light-responsive melanopsin-containing ganglion cells. DAPI-stained nuclei are shown in blue in the coronal section and the autofluorescent debris zone (dz) is indicated. (B) Light-induced c-Fos activation in the transplanted eye of RCS rats is preferentially preserved in the dorsal retina (the region of the transplant), corresponding with preservation of photoreceptors (ONL) by the iPS-RPE graft. No such preservation is observed in the ventral retina of the transplanted animal. Scale bars: Coronal sections, 50 µm; whole-mount images of the inner nuclear and retinal ganglion cell layers, 200 µm.

To assess the topography of retinal function in grafted versus non-grafted eyes we employed functional anatomy to look at the activation of inter-neurons of the inner nuclear layer (INL). This was achieved by examining the light-induced expression of c-Fos, a member of the immediate early gene family. Light is known to induce nuclear c-Fos expression in the INL of the mouse retina, however this response is reduced in a mouse model of photoreceptor degeneration[34]. In the normal, non-dystrophic rat retina, the ONL was present and there was clear activation of c-Fos in the INL in response to light, with a complete absence of staining in the dark (Fig. 9A). Only occasional foci of c-Fos positive cells were detectable in the INL of the non-transplanted dystrophic retina where the ONL consisted of a single layer. Following transplantation of iPS-RPE cells, there was a clear preservation of light-induced c-Fos activation in the INL beneath regions of photoreceptor preservation and in the ganglion cell layer (GCL), the output layer of the retina. The distribution of occasional light-activated c-Fos cells observed in the GCL of non-transplanted dystrophic rats matches that reported for intrinsically light-responsive melanopsin ganglion cells[35]. Importantly, in grafted animals, preservation of the ONL and light-induced c-Fos expression in the INL and GCL was restricted to the dorsal retina, the region where iPS-RPE cells had been injected (Fig. 9B).

## Discussion

The successful differentiation of iPS cells into RPE represents a significant advance in the search for a potential cell source for the treatment of human neural retinal diseases. iPS(IMR90)-3 cells readily differentiate into RPE cells and the differentiation protocol used in this paper is a highly efficient method of producing multiple confluent flasks of highly enriched pigmented cells. An efficient initial RPE differentiation protocol is essential for the production of cells for use in any therapeutic application since repeated passaging of RPE results in phenotypical and morphological changes associated with dedifferentiation[36], [37]. The RPE cells derived from iPS(IMR90)-3 cells have been well-characterized here and satisfy many of the known criteria of RPE cells, including protein expression, cellular pigmentation and polarization. These properties are similar to those observed in cultured HESC-derived RPE[5]–[7], [9]. iPS-RPE also down-regulate the embryonic transcription factors originally used to induce pluripotency from the somatic cell[18], suggesting differentiation away from the initial iPS phenotype.

Critical functions of RPE cells include the maintenance of photoreceptor cell integrity by phagocytosing debris shed by the retina each day and epithelial transport of molecules and metabolic waste[38]. Functional properties of RPE cells are also observed in iPS-RPE *in vitro*, including the transport of fluid across cells, as indicated by the formation of blister-like domes in the monolayer[39]. Similar to HESC-derived RPE, iPS-RPE can phagocytose photoreceptor outer segments from isolated preparations and porcine retina explants[8]. A recent paper has also described the *in vitro* phagocytic properties of iPS-RPE cells[25] derived using an alternative culture protocol [5], however, the *in vivo* function of these cells has yet to be tested.

After 8 days in the sub-retinal space of the dystrophic RCS rat, iPS-RPE cells are capable of phagocytosing host photoreceptor outer segments. This evidence of *in vivo* phagocytosis is characterised by the presence of an exclusive outer segment marker, rhodopsin, within the cytoplasmic compartment of cells labelled with HSM. These data suggest that human iPS-RPE cells are able to contribute to host photoreceptor cell integrity by removing retinal debris at this time-point.

iPS-RPE cells were transplanted into dystrophic RCS rat eyes at three weeks of age, a time when retinal abnormalities are first observed. The progression of retinal dystrophy in the RCS rat is such that by 13 weeks post-graft most of the ONL has disappeared [33] and the photoreceptor outer segment layer is reduced to a debris zone[40], a finding we observed in dystrophic controls. The preservation of these layers 13 weeks after iPS-RPE cell injection suggests that the transplantation of these cells preserves retinal structure. At this stage we also performed analysis of visual function. Visual acuity tests showed that the performance of animals receiving iPS-RPE transplants was significantly better than sham-operated and control animals. We also identified the presence of functional neuronal circuitry in the retina after iPS-RPE transplantation as indicated by the light induced c-Fos response in the INL and GCL of the neural retina. This is the first time that that this assay has been used to ascertain retinal function after cellular therapy in a retinal degenerate animal. Importantly, the preservation of light-induced c-Fos expression was restricted to the dorsal retina, surrounding the region of iPS-RPE cell injection only, suggesting preservation of localised neural activation corresponding to the histology of rhodopsin expression. Previous studies, using electroretinography to assess function following cell transplantation into the RCS rat have only been able to demonstrate global activity across the retina [6], [41]–[43].

The absence of iPS-RPE cells in the subretinal space at the time of functional assessment (13 weeks) indicates that the significant benefits observed could not be wholly attributed to the donor cells. Although all animals were maintained on an oral immunosuppression drug, ciclosporin, throughout the experiment, this was not sufficient to sustain iPS-RPE cell survival. These findings are in agreement with previous studies that show that xenografts can be compromised[4], [44] even after triple immune suppression[45]. Our analysis suggests that loss of transplanted cells is associated with infiltration of the subretinal space by macrophages/microglia. The large pigmented CD68-positive cells observed in the subretinal space at 13 weeks are likely to be macrophages/microglia filled with melanin[46], [47] from the transplanted human iPS-RPE cells. The presence of rhodopsin within the macrophages/microglia could explain some of the behavioural and functional benefits observed, since clearance of outer segment debris by these cells in the subretinal space could also contribute to photoreceptor cell survival. This conclusion has been implied previously in a study which suggests that macrophage infiltration in response to the trauma of retinal detachment after saline injection, contributes to extend the longevity of photoreceptor cells in the RCS rat[48]. Our finding highlights a key aspect of cellular transplantation not fully addressed in many short or long-term studies of RPE transplantation: the host inflammatory/immune response to the xenograft and its indirect role in the preservation of the retina. Importantly, we show that the presence of pigmented cells within the subretinal space does not necessarily reflect survival of transplanted cells. We suggest correct identification of the origin of these cells (using human specific markers, which define cell membranes) is essential in order to distinguish viable donor cells from host inflammatory cells which have engulfed transplanted cells. Identification using pigmentation alone is not sufficient.

The increased visual acuity and outer nuclear layer preservation observed in iPS-RPE injected animals could also be due to a neuroprotective effect produced by the donor cells. RPE are known to secrete neurotrophic growth factors such Glial cell derived neurotrophic factor (GDNF), Brain derived neurotrophic factor (BDNF)[49], PEDF[50] and bFGF[51]. As these factors can exert protective effects on retinal neurons[52]–[56] and neurons in other neurodegenerative disease models [49], [57], [58], it seems likely that these substances may contribute to the latent photoreceptor cell survival we observe in the dystrophic retina. The fact that the area of preservation extends beyond the borders of the graft suggests that a neuroprotective effect may also be involved. Release of growth factors can occur as a result of surgery but the effects are of shorter duration.

The major limitation of this study is the rejection of human iPS-RPE after transplantation into the RCS rat. RPE cells derived from HESC show long-term viability *in vivo*, since in a previous study we have shown that HESC-derived RPE can survive in the RCS subretinal space for up to 10 weeks[9], whilst a recent study has indicated survival of cells for up to 30 weeks[59]. The embryonic origin of HESC-derived RPE may reflect a more immune privileged cell type in comparison to iPS-RPE, which contributes to their longer-term survival after xenografting. HESC have been shown to have reduced immunogenicity, expressing low levels of MHC-I and MHC-II in both the undifferentiated and dedifferentiated state, and to possess an adaptive mechanism to immune responses[60]–[62]. As yet the immunogenic profile of iPS or iPS-derived cells has not been described. Thus, the RCS rat retina offers a useful model for the short-term analysis of iPS-RPE cell function *in vivo*, but the donor cell loss due to host macrophage infiltration of the xenograft indicates that additional modification may be necessary to promote long-term donor cell survival in this animal model system.

The major benefit of using iPS cells to treat AMD is that developing a patient specific therapy may help to eliminate the problems associated with immune rejection. Proof of concept for the therapeutic use of a patient's own iPS-derived RPE lies in current clinical treatments for AMD. Although complicated, surgical procedures such as the autologous transplantation of peripheral RPE to the macular region[63]–[66] and macular translocation, where the neural retina is detached and the fovea relocated to a less diseased area of RPE[67]–[69], have been shown to stabilize visual acuity in AMD patients. Consequently, although iPS-RPE may still carry the same genetic defect responsible for AMD in the patient, the fact that these cells have not been diseased by age, like macular RPE, suggests that they could still be used as a viable therapeutic. iPS cell therapy might also be useful in patients with genetic diseases, such as Leber's congenital amaurosis where transplantation could be combined with gene therapy to correct genetic defects inherent to the patients' own RPE cells. Alternatively iPS cells derived from a tissue-matched healthy sibling may also be useful[70]. iPS-RPE may also provide a useful *in vitro* model system in which to study the pathogenesis of human RPE-linked diseases and identify novel molecular/biochemical therapeutic targets.

While this particular line of iPS-RPE cells could not be used as a direct therapeutic due to viral insertions of pluripotency genes, the recent advances in iPS cell reprogramming technology, including the use of small molecules[71]–[73], *piggyBac* transposition[74], [75], non-integrating episomal vectors[76] and manipulation of endogenous transcription factors[77] should eliminate the risks associated with integration of stem cell genes into the genome. Furthermore, the finding that blood cells can be used to derive iPS cells[78] may remove the need for invasive patient biopsies required for the collection of somatic cells and accelerate the ethical production of stem cell-derived tissue for therapeutic use.

## Supporting Information

Table S1 Primer pairs used for RT-PCR and quantitative PCR. Amplicon size is in base pairs (bp).
(0.09 MB DOC)

Movie S1 Phagocytosis of fluorescently labelled photoreceptor outer segments (green) by iPS-RPE cells in vitro. The apical surface of the RPE cells is identified by ATP1B1 (red).
(0.50 MB MOV)

Movie S2 Visual acuity testing of the non-transplanted eye. No head-tracking response is observed in the 16-week old dystrophic RCS rat during optokinetic testing of the right, non-transplanted, eye.
(1.80 MB MOV)

Movie S3 Visual acuity testing of the iPS-RPE transplanted eye. A head-tracking response is observed in the dystrophic RCS rat during optokinetic testing of the left eye, 13 weeks after iPS-RPE cell transplantation.
(1.90 MB MOV)
