# Supplementary material for: Protective Effects of Human iPS-Derived Retinal Pigment Epithelium Cell Transplantation in the Retinal Dystrophic Rat
Source: PLoS One. 2009 Dec 3;4(12):e8152. doi: 10.1371/journal.pone.0008152 (PMC2780911; doi:10.1371/journal.pone.0008152)
Supplement: Table S1 — Primer pairs used for RT-PCR and quantitative PCR. Amplicon size is in base pairs (bp). (0.09 MB DOC) [file pone.0008152.s001.doc]

Table S1

| **Gene** | **Accession** |  | **Sequence (5’-3’)** | **Size (bp)** | **Ta** |
| --- | --- | --- | --- | --- | --- |
| RT-PCR Primers | | | | | |
| *Best1* | NM_004183.3 | F | AGGTCGAATCCGGGACCCTA | 226 | 64 |
|  |  | R | GCACAACGAGGTCCAGCTCA |  |  |
| *Fak* | AY323812 | F | ATGTGACGGGCCTGGTGAAAGG | 160 | 58 |
|  |  | R | TGGGTGCTGGCTGGTAGGAG |  |  |
| *Krt8* | NM_002273 | F | AAGGATGCCAACGCCAAGTT | 214 | 60 |
|  |  | R | CCGCTGGTGGTCTTCGTATG |  |  |
| *Lrat* | NM_004744 | F | GAATGAGGCATGGACCTGTT | 203 | 52 |
|  |  | R | TCCTGCCCAAAATCTGTTTC |  |  |
| *MerTK* | NM_006343 | F | GTGAGGCAGCGTGCATGAAAG | 95 | 58 |
|  |  | R | GGGCTTTGGGATGCCTTGAG |  |  |
| *Pedf* | NM_002615 | F | AGCTCGCCAGGTCCACAAAG | 222 | 60 |
|  |  | R | TGGGCAATCTTGCAGCTGAG |  |  |
| *Pmel17* | NM_006928 | F | GTGGTCAGCACCCAGCTTAT | 233 | 52 |
|  |  | R | GAGGAGGGGGCTATTCTCAC |  |  |
| *Rlbp1* | NM_000326 | F | GCTGCTGGAGAATGAGGAAACTC | 173 | 52 |
|  |  | R | GGCTGGTGGATGAAGTGGAT |  |  |
| *Rpe65* | NM_000329 | F | GCCCAGGAGCAGGACAAAAG | 246 | 52 |
|  |  | R | GCGCATCTGCAAGTTAAAACCA |  |  |
| *Tyr* | NM_000372 | F | TGCCAACGATCCTATCTTCC | 316 | 52 |
|  |  | R | GACACAGCAAGCTCACAAGC |  |  |
| Quantitative PCR Primers | | | | | |
| *Oct3/4* | NM_002701 | F | CTGTCTCCGTCACCACTCTG | 168 | 56 |
|  |  | R | TGTGTTCCCAATTCCTTCCTTAG |  |  |
| *Nanog* | NM 024865.2 | F | CCCTCCTCCCATCCCTCATAG | 116 | 56 |
|  |  | R | TCGCTGATTAGGCTCCAACC |  |  |
| *Mitf* | NM 198759.1 | F | TTGTCCATCTGCCTCTGAGTAG | 87 | 56 |
|  |  | R | CCTATGTATGACCAGGTTGCTTG |  |  |
| *Otx2* | NM 021728.2 | F | ACCTTGAACTCCACCTCTGC | 172 | 56 |
|  |  | R | GCTTCTCTTCTCTGACTCTCTTTG |  |  |
| *RPE65* | NM 000329.2 | F | TACAGAAAGCACTGAGTTGAGC | 153 | 56 |
|  |  | R | CCATTTAGTAAGTCCACATTCATTTCC |  |  |
| *Tyr* | NM 000372.4 | F | GTGTAGCCTTCTTCCAACTCAG | 125 | 56 |
|  |  | R | GTTCCTCATTACCAAATAGCATCC |  |  |
| *Pmel17* | NM 006928.3 | F | GTTGATGGCTGTGGTCCTTG | 95 | 56 |
|  |  | R | CAGTGACTGCTGCTATGTGG |  |  |
| *PEDF* | NM 002615.4 | F | TATCACCTTAACCAGCCTTTCATC | 82 | 56 |
|  |  | R | GGGTCCAGAATCTTGCCAATG |  |  |
| *GAPDH* | NM 002046.3 | F | AGCAAGAGCACAAGAGGAAGAG | 175 | 56 |
|  |  | R | GAGCACAGGGTACTTTATTGATGG |  |  |
| *PPIA* | NM 021130.3 | F | CCTCCCAAACTGCTGAGATTAC | 101 | 56 |
|  |  | R | TGCTTCCCTCAAGAACATCAAC |  |  |
| *HMBS* | NM 000190.3 | F | TGCTATCTGGGGAGTGATTACC | 146 | 56 |
|  |  | R | GGCTGTTGCTTGGACTTCTC |  |  |
| *GPI3’* | NM 000175.2 | F | GGACCACGAGCCCTTAGC | 99 | 56 |
|  |  | R | AACACTTCAGCCAATTCTAACAC |  |  |
| GPI 5’ | NM 000175.2 | F | CGTCATCAACATTGGCATTGG | 85 | 56 |
|  |  | R | GGGACCTCCTGAAGAGTATGG |  |  |
